# “Nothing to Lose, Absolutely Everything to Gain”: Patient and Caregiver Expectations and Subjective Outcomes of Deep Brain Stimulation for Treatment-Resistant Depression

**DOI:** 10.3389/fnhum.2021.755276

**Published:** 2021-09-29

**Authors:** Cassandra J. Thomson, Rebecca A. Segrave, Paul B. Fitzgerald, Karyn E. Richardson, Eric Racine, Adrian Carter

**Affiliations:** ^1^School of Psychological Sciences, Turner Institute for Brain and Mental Health, Monash University, Clayton, VIC, Australia; ^2^Epworth Centre for Innovation in Mental Health, Epworth Healthcare, Camberwell, VIC, Australia; ^3^Department of Psychiatry, Central Clinical School, Monash University, Melbourne, VIC, Australia; ^4^Pragmatic Health Ethics Research Unit, Institut de Recherches Cliniques de Montréal, Montreal, QC, Canada; ^5^Department of Medicine and Social and Preventive Medicine, Université de Montréal, Montreal, QC, Canada; ^6^Medicine and Biomedical Ethics Unit, Department of Neurology and Neurosurgery, McGill University, Montreal, QC, Canada

**Keywords:** deep brain stimulation (DBS), neuromodulation, depression, informed consent, expectations, subjective outcomes, ethics, neurotechnology

## Abstract

**Background:** How “success” is defined in clinical trials of deep brain stimulation (DBS) for refractory psychiatric conditions has come into question. Standard quantitative psychopathology measures are unable to capture all changes experienced by patients and may not reflect subjective beliefs about the benefit derived. The decision to undergo DBS for treatment-resistant depression (TRD) is often made in the context of high desperation and hopelessness that can challenge the informed consent process. Partners and family can observe important changes in DBS patients and play a key role in the recovery process. Their perspectives, however, have not been investigated in research to-date. The aim of this study was to qualitatively examine patient and caregivers’ understanding of DBS for TRD, their expectations of life with DBS, and how these compare with actual experiences and outcomes.

**Methods:** A prospective qualitative design was adopted. Semi-structured interviews were conducted with participants (six patients, five caregivers) before DBS-implantation and 9-months after stimulation initiation. All patients were enrolled in a clinical trial of DBS of the bed nucleus of the stria terminalis. Interviews were thematically analyzed with data saturation achieved at both timepoints.

**Results:** Two primary themes identified were: (1) *anticipated vs. actual outcomes*, and (2) *trial decision-making and knowledge*. The decision to undergo DBS was driven by the intolerability of life with severe depression coupled with the exhaustion of all available treatment options. Participants had greater awareness of surgical risks compared with stimulation-related risks. With DBS, patients described cognitive, emotional, behavioral and physical experiences associated with the stimulation, some of which were unexpected. Participants felt life with DBS was like “a roller coaster ride”—with positive, yet unsustained, mood states experienced. Many were surprised by the lengthy process of establishing optimum stimulation settings and felt the intervention was still a “work in progress.”

**Conclusion:** These findings support existing recommendations for iterative informed consent procedures in clinical trials involving long-term implantation of neurotechnology. These rich and descriptive findings hold value for researchers, clinicians, and individuals and families considering DBS. Narrative accounts capture patient and family needs and should routinely be collected to guide patient-centered approaches to DBS interventions.

## Introduction

There is a pressing need for novel and effective treatments for people living with treatment-resistant depression (TRD). Approximately one-fifth of all people who experience depression will not respond to existing evidence-based therapies (Fava, 2003). Deep brain stimulation (DBS) is a potential treatment for depression currently being investigated. Primary outcome measures used in clinical trials of DBS for depression include the Hamilton Rating Scale for Depression (Hamilton, 1960) and Montgomery-Åsberg Depression Rating Scale (Montgomery and Asberg, 1979). While valuable for assessing subjective changes in depression symptoms as defined in the Diagnostic and Statistical Manual of Mental Disorders (DSM-5), these measures do not provide a comprehensive picture of the intervention’s overall impact and often do not fully capture participants’ beliefs about the benefit they have gained (de Haan et al., 2015; Mayberg, 2018). What “well” looks like and what is considered a “success” is also highly specific to each individual (Fins et al., 2017).

Qualitative investigations with those who undergo DBS is one method for gaining a more holistic and comprehensive understanding of intervention outcomes. Despite the growing recognition and need for patient-centered care and the elevation of patient voices within medical research and clinical practice (Greenhalgh et al., 2016; Sidhu et al., 2017; Braun and Clarke, 2019), few qualitative studies with this population have been conducted. Acquiring qualitative data from health care recipients and lived experience experts (e.g., patients, caregivers) is vital for improving the translation of clinical research outcomes into standard practice and health care (Institute of Medicine, 2001). In addition to highlighting an intervention’s successes and failures, qualitative data can reveal the meaning and significance of changes experienced by patients, for both themselves and those closest to them.

Patient expectations about the likely benefit of undergoing DBS can affect clinical outcomes (Okun and Foote, 2004; Maier et al., 2013), as well as raise challenging ethical questions when trialing DBS for mental illnesses, such as TRD (Bell and Racine, 2013). A handful of qualitative studies have explored the relevance of questions including whether individuals with severe and refractory depression have the capacity to consent to an experimental procedure and whether their decision to participate is motivated by unrealistic expectations of personal benefit (Christopher et al., 2012; Fisher et al., 2012). Fisher and colleagues interviewed 31 people enrolled in two DBS for depression trials and assessed their decision-making and capacity to consent using semi-structured interviews. All participants demonstrated intact capacity to consent; however, therapeutic misconception was present amongst some (i.e., participants viewed the study’s purpose as specifically helping their mental health rather than producing generalizable knowledge and underrated risks and overrated likelihood of personal benefit). The authors note that similar degrees of therapeutic misconception are represented in other clinical and non-clinical populations; therefore, they concluded that people with TRD do not appear uniquely susceptible to therapeutic misconception. Based on these results, informed consent processes for the two DBS trials were considered by the authors as sufficiently robust (Christopher et al., 2012).

Post-DBS qualitative data provides some different perspectives on these ethical issues. Klein et al. (2016) conducted focus groups and interviews with 15 recipients of DBS for either TRD and obsessive-compulsive disorder (OCD), exploring their attitudes toward emerging closed-loop DBS systems. In doing so, participants reflected on their own experiences of enrolling in an experimental DBS trial. Some perceived that depression-related cognitive and emotional vulnerabilities impacted their comprehension of information and how they evaluated the associated risks (“I could have cared less about the risks” p. 145). Some recalled a sense of desperation to rid themselves of depression with which high hopes and unrealistic expectations emerged. These findings suggest that nuanced consideration is needed when it comes to the process of conducting robust informed consent with this population.

The current study extends upon the existing research by exploring how these important pre-intervention ethical issues (e.g., decision-making capacity, awareness of risks/benefits, and expectations) are related to participants’ post-intervention outcomes and experiences. Caregivers (spouses, family members) were also included in the study. Caregivers have remained absent within psychiatric DBS research despite the fact they often play a significant role during all stages of clinical trial participation (e.g., decision-making, attending medical and research-related appointments, observing changes in the participant after DBS) (Klein et al., 2016; Thomson et al., 2019a). The purpose of the current study was to gain in-depth knowledge and insight into the experience of preparing for, and living with, DBS for TRD. To achieve this, semi-structured interviews were conducted with key stakeholders (e.g., patients and caregivers) before and after DBS. More specifically, the study aimed to examine: (1) factors influencing the decision to pursue DBS; (2) participants’ knowledge and understanding of DBS for TRD, including potential risks and benefits; (3) expectations held by patients and caregivers prior to DBS; (4) the subjective outcomes of the intervention; and (5) how outcomes compare with original expectations.

## Materials and Methods

This exploratory study employed a prospective qualitative design and iterative thematic analysis approach. This article reports on the experiences of a subset of participants enrolled in a clinical trial of DBS for TRD (Australian New Zealand Clinical Trials Registry: ACTRN12613000412730)^1^. Separate and non-overlapping findings from the current sample examining personal and relational changes following DBS are reported elsewhere (Thomson et al., unpublished).

### Participants

A consecutive sampling approach was used to recruit participants actively enrolled in the clinical trial who were awaiting surgery. These participants met Stage V criteria for TRD according to the Thase and Rush (1995) classification. This is the most severe classification with individuals failing to respond to adequate courses of all evidence-based therapies, including pharmacotherapy (all antidepressant classes and combination/augmentation strategies), psychotherapy (including but not limited to cognitive behavioral therapy) and non-invasive brain stimulation [electroconvulsive therapy (ECT), transcranial magnetic stimulation]. All participants had presented to the Victorian Mental Health Tribunal and received approval to undergo DBS for depression. The first-author (CT) recruited DBS candidates and their respective caregivers to the current study, providing them with verbal and written study information. All who were approached, agreed to participate in the study. The sample consisted of six DBS candidates and five caregivers (see Table 1). One candidate did not have a caregiver and participated independently.

**TABLE 1:** Participant demographic information.

**Variable**	**Patients (*n* = 6)**		**Caregivers (*n* = 5)**	
Gender	Women = 5	Men = 1	Women = 2	Men = 3
Relationship type			Spouse = 4	Parent = 1
Work status	Unemployed = 5	Volunteer = 1	Employed = 4	Retired = 1
	**Mean (*SD*)**	**Range**	**Mean (*SD*)**	**Range**
Age (years)	52.3 (16.9)	26–73	59.6 (11.7)	45–75
Education (years)	14.3 (2.1)	12–17	14.4 (1.3)	12–15
Time since diagnosis (years)	18.3 (12.3)	8–42		
Relationship length (years)			37 (12.3)	24–50

### Procedure

Semi-structured interviews were conducted either face-to-face (at participants’ home, research center) or via telephone/video-conference (for participants living interstate). Patients and caregivers were interviewed separately to allow for open discussion. Interviews were conducted by the first-author (CT), a female psychologist with training in qualitative methods and experience interviewing DBS patients, caregivers and clinicians (Thomson et al., 2019b, 2020). Pre-surgery interviews (*n* = 11, *M* = 46 min, range = 34–58) occurred 3–15-weeks prior to surgery. These interviews explored participants’ decision-making process, awareness and understanding of associated risks and benefits, and expectations and beliefs about potential outcomes (see Supplementary Material for interview schedules). Further probing questions were asked to elicit greater depth of information and responses were reflected back to participants to ensure interviewer understanding. Participants then underwent DBS surgery, with electrodes implanted in the bed nucleus of the stria terminalis. Surgery and recovery from surgery was medically uncomplicated for all participants. After a recovery period, participants commenced a randomized schedule of active and sham (control condition) stimulation settings, to which they were blinded. Over 5 months, five stimulation settings were trialed: one inactive, two low level (2 volts), and two moderate level (4 volts). Following this, stimulation continued in an open-label manner, with settings optimized according to each individual. Post-surgery interviews (*n* = 10, *M* = 55 min, range = 36–86) occurred 9–11-months after surgery and approximately 3-months into the optimization phase. These interviews explored participants’ experiences living with DBS, their subjectively perceived outcomes, and reflections on their pre-surgery beliefs and expectations. One candidate did not complete a postoperative interview as it was deemed too burdensome. Field notes were maintained and regular debriefing occurred between co-authors (CT, AC, RS). All interviews were digitally recorded and transcribed verbatim by a professional transcription service. Transcriptions were reviewed for accuracy and de-identified (by CT).

### Qualitative Analysis

An iterative thematic analysis approach was chosen as it is suitable for exploring a single phenomenon (e.g., undergoing DBS) from different perspectives and can be used to highlight similarities, differences and inconsistencies in perspectives across time (Braun and Clarke, 2006). The analysis was conducted within a realist paradigm, which assumes a direct relationship between language and meaning or experiences. Therefore, participants’ language was assumed to represent the reality of their lives and reflect the meaning they assign to their experiences. The analysis and interpretation were conducted with a psychological lens, although the process of peer debriefing allowed input from different perspectives and disciplines (neuroethics, social science, neuropsychology) (Yilmaz, 2013).

Transcript data was imported into and organized using NVivo 12 software (QSR International Pty Ltd., Doncaster, Australia). Thematic analysis was conducted according to the six-step process outlined by Braun and Clarke (2006). This involved: familiarization through repeating listening and reading of interviews, initial generation of codes, searching for themes, reviewing and refining themes, defining and naming themes, and reporting using representative quotes with pseudonyms to protect confidentiality. The analysis process was iterative and inductive (data-driven), aligning with the “codebook” approach to thematic analysis (Braun and Clarke, 2020). Cross-coding was conducted on a subset (6) of interviews (by CT, AC, RS), with discussions held amongst the coding team to develop and refine a coding structure. All interviews were subsequently coded by the first-author and interviewer (CT). Data saturation, the point at which no novel themes were identified in analysis, was reached at interview 9 of the pre-DBS interviews (total of 11) and 9 of the post-DBS interviews (total of 10). The “Consolidated Criteria for Reporting Qualitative Research” (COREQ) was used to support transparent and comprehensive methodological reporting (Tong et al., 2007).

## Results

The thematic analysis revealed two primary themes that are presented within the current article (Table 2). Primary themes developed longitudinally with secondary themes reflecting specific timepoints (pre-/post-DBS). Patient and caregiver perspectives are represented across all themes, with example quotes presented in Tables 3, 4.

**TABLE 2:** Thematic analysis matrix.

	**Anticipated vs. actual outcomes**	**Trial decision-making and knowledge**
Pre-DBS	• Anticipated responses to stimulation • Balancing hopes and expectations • Approaches to aid recovery	• Process of decision-making • Awareness and weighing of risks • Knowledge and preparedness
Post-DBS	• “A roller coaster ride” • Responses to stimulation adjustment • Reflections on expectations: “work in progress”	• Reflections on preparedness • Knowledge transfer • No regrets

**TABLE 3a:** Anticipated vs. actual outcomes—pre-DBS.

**Anticipated responses to stimulation**
Patient 1: *My understanding is that most people can’t actually differentiate between when the stimulation is on or off to start with and you would therefore not be likely to differentiate between one set of stimulation parameters and another except in the case that a particular set of stimulation parameters did in fact provide a benefit.*
Patient 4: *I’ve read of patients where they’ve basically had it switched on and it’s worked straightaway and they’ve felt better straightaway…I’m probably being unrealistic but I would expect to know within a day or two if it was going to be the right frequency for me.*
*Caregiver 3: For me, a number of weeks probably would be quickly [for a response]…I’m not expecting instant results but I’m not sure if it’s going to be weeks or months before we get to that stage.*
**Balancing hopes and expectations**
Patient 3: *An expectation and a hope are very different things, in my mind. I don’t expect anything, because when you’ve been in my position for as long as I have, you can’t expect anything. They tell you to expect to get better with every new medication they put you on, when they augment your medication, when they try a new therapy. You learn not to expect anything, because you just become disappointed and end up plunging further in. Whereas if you don’t ever hope, you don’t get knocked down, essentially. There’s a chance, but it’s a very detached kind of feeling. It’s very clinical…there’s a chance and I’ll take that chance.*
Patient 4: *I expect to get better. I expect to recover…I expect to go back to work. I expect to go back to my social life. I expect to be able to travel…I expect to be happy and I expect not to wake up every day and wish I was dead…I expect to not wake up and start crying and say god help me through another day…Yeah but maybe I’m being a bit overoptimistic about the whole procedure but I just need to think of it in very, very positive terms because it’s such a big thing to go through…I can’t not do that because I’ll just give up. I just couldn’t go through with it if I didn’t think it was going to be very beneficial.*
Patient 6: *I’m not expecting—nobody’s telling me I’m going to get a total recovery. But 50 per cent of where I used to feel, 20 years ago. More energy. More interest. More just more, more, more of what’s surrounding me. More of the family. That would do me.*
Caregiver 6: *We’ve been there and we’ve hoped so many times, had it dashed that many times, I don’t know if…I’ve got it in me to hope for a cure anymore. It’s an awful thing to say, isn’t it?… Well, you work up and you get knocked out again. So, much better to be delightfully surprised than to be up there and come crashing down again…Anything has to be an improvement. I’d accept crumbs, but a big slice of the cake would be better [laughs]. I’ll take anything.*
**Approaches to aid recovery**
Patient 3: *I’m under no illusion that I probably will be on medication for the rest of my life…I never found talking based therapies of any use, so I wouldn’t actively pursue that…[I’m] wanting to hopefully have a bit more of a social network…Hopefully being able to join a sport…one, to make friends, but two, the physical activity does help with stress reduction as well as depression…That would be lifestyle things. But as far as having counselling, no.*
Patient 5: *[Continuing] probably what I’m doing now, I think. I have, as I say the psychiatrist and she’s very supportive too, but then the psychologist, she’ll work on different things I’m going through at the time…Yeah, unless there’s some magic, and it’s really good, well I would be continuing them.*
Caregiver 5: *We realise that she’s still going to have like five months of trialling the different modes…She needs to keep on seeing a psychiatrist and counsellor…and we know that the medication is going to keep on going for some time until we find out what the results are going to be. So, we realise there’s still going to be a lot of support and a lot of time and effort put into it.*

**TABLE 3b:** Anticipated vs. actual outcomes—post-DBS.

**“A roller coaster ride”**
Patient 4: *It’s like you’re on a roller coaster, and you think you’re better and you get so happy because you think you’re better and then you crash again…That’s extraordinarily disappointing…It’s very subtle, and I really expected it would be boom, boom, boom and you’re better, I didn’t think it was going to take this long. They probably don’t need to tell you that in the beginning, do they?*
Patient 5: *[It’s been] very changeable…It’s really constantly changing…It depended on the adjustment…Initially I’d get very hot…one time I got very anxious…[Or] I might have an initial time where—a bit more energy and probably a little bit more interest in things but it wouldn’t last.*
Caregiver 2: *I think that the differences are quite pronounced. It’s worked and it’s definitely transforming, and it’s going to take some time for her to work through it…We’re getting there, but it doesn’t come without its own set of drama.*
**Responses to stimulation adjustment**
Patient 1: *I would say with the last setting…there’s perhaps some improvement in my sleep. I guess some small and very fleeting kind of perception of improvement in mood generally and that kind of pervasive pessimism. It’s kind of small and very fleeting.*
Patient 6: *That [adjustment] was really, really, really dramatic. It really was. I couldn’t stop talking. I was talking to every man and his dog. I was wired. It was very frightening…No, this was all way over the top.*
Caregiver 4: *[Patient name] had a very bad experience during the trial period where she reacted badly…as in setting that didn’t suit. Generally, when there is an adjustment, there will be a little bit of time where she—I don’t know how to really describe it, but anyway it upsets her system somehow, her body, her mind.*
**Reflections on expectations: “work in progress”**
Patient 2: *I can see it getting to the top. It’s taken a little bit of time to get used to being happy and all the emotions that go with that…I was right down at the bottom and now I’m getting closer to the top—and liking it.*
Caregiver 3: *I think it’s still a work in progress, yeah. The fact that I’ve said that there have been times where she has been motivated and what have you…I do think that we can see that the stimulator is having an effect. Sometimes the effect may be a little bit over the top [laughs] but yeah, I’m still hopeful that we will get a result.*
Caregiver 5: *[I’m] still hopeful…because it seems to be…a number of options…Four by eight vaults by four by eight vaults, well it’s a lot of options…I’m probably an optimist and hopefully there’s something there that means it’s going to help. How long do you keep on doing it for, I don’t know?*

**TABLE 4a:** Trial decision-making and knowledge—Pre-DBS.

**Process of decision-making**
Patient 1: *Initially, I spent a lot of time reading about it to try to understand the rationale behind it…the model of depression which underlies this as a treatment because it’s quite a different model from most biochemically focused models. I spent some time reading the peer review literature reporting on various types of clinical trials elsewhere. Simultaneously with that, I had had many discussions with my own psychiatrist about what other options there might be in a context where you’ve tried lots and lots and lots of treatments with no real benefit. I did spend quite some time weighing up the costs and benefits…both in the sense of risk but also…the effort required to engage with this.*
Patient 4: *I really don’t like the idea of your brain being operated on but if you’ve decided this is what you need to do—and I would sum it up in one sentence. I would do this in a heartbeat rather than live with the agony of depression for the rest of my life. And you can quote that one. It’s a good quote…I really don’t have any other options left. I’ve searched the whole world for something and there is none. There’s always an answer to a problem but I looked pretty hard and I think that this is probably my best solution now.*
Patient 5: *Over all the time there’s been two people that said to me ‘give it a go,’ other than that people have been non-committal, which I can understand. It’s a huge thing to do, and it’s a huge commitment and everything. Yes, so it is my decision but it’s a very lonely place at times when you’re just on your own wondering what you should do.*
Caregiver 2: *We’re both aware it’s very much a research project. She’s actually said on a couple of occasions she wants to help that cause. Not only does she want herself to get better but she wants to further that science so other people can benefit from it. So, she’s more than willing to go through that.*
**Awareness and weighing of risks**
Patient 1: *I’m conscious that there are some risks associated with the surgery, that are potentially catastrophic but with a very low probability…There are risks associated with anaesthetic in general, there’s a risk of stroke, there’s a risk of…a small bleed in the brain. It would not be defined as a stroke but might nonetheless cause some impairment. So, it might be transitory or permanent. There’s a risk of infection of the wound, there’s a risk of infection, less commonly, of the brain itself. A stroke itself could have a whole range of effects in terms of paralysis and language function.*
Patient 2: *The process of having the ECTs and general anaesthetics and that…it’s sort of made the process [of DBS] less scary because I’ve gone through all of that and having the ECT and all the risks of that was probably the same as having the DBS. The only thing is, if the DBS works then I don’t have to have any more of the ECTs.*
Interviewer: *What about after surgery, when you’ve got the stimulation running, do you know about some of the side effects associated with the stimulation?*
Patient 3: *Not really. I imagine, short of recovery, once it’s in and it’s settled that you wouldn’t really be aware that it was there at all.*
**Knowledge and preparedness**
Patient 4: *Yeah, they’ve given me huge amounts of information. I’ve been very well informed. Over-informed to the point where you’re just like I don’t want to know about it, just do it.*
Caregiver 2: *We’ve had a couple of different information packs sent to us from the team…We’ve had probably I’d say at least 40 discussions about it with various people on the team…Yeah, we’ve had plenty of information on it…We can’t say we weren’t forewarned!*
Caregiver 3: *I’m probably not at all informed as to what happens after the surgery…I know she’s going to have to go back a few times to…change the settings and that kind of stuff but I haven’t been given any information on what to expect really, after the surgery. I think [patient] has been given that info but I haven’t really been given too much on that side of things…No doubt I will get more information on it as we go down the track but yeah, I…don’t know exactly what to expect, going forward.*

**TABLE 4b:** Trial decision-making and knowledge—post-DBS.

**Reflections on preparedness**
Interviewer: *Do you think that looking back that you felt fully informed of all the potential risks and side effects associated with DBS?*
Patient 4: *I actually don’t remember. I don’t remember, because when you’re depressed your memory was rubbish. You’re not interested…and they’re telling you, you could die, and you go actually I don’t care, I want to die anyway, so what difference does it make? No, I mean they probably did inform me, I’m sure legally they had to, but I wasn’t listening.*
Interviewer: *[…] Has there been anything that’s come as a surprise through this process? Anything that you perhaps didn’t expect coming in to it?*
Patient 4: *Absolutely, I didn’t realise it was going to be such a tough journey finding frequencies and such a long journey, and such a rollercoaster…thinking “I’m on it, I’m getting better, this is great,” and then two days later you go bang and you’re back to where you are…[I] didn’t know it was going to be so hard, that was a big surprise, and I didn’t need to know it beforehand. Because I couldn’t have coped. What you don’t know can’t hurt you sometimes.*
Interviewer: *Yeah, so do you think if you’d been told that prior, that it could be very variable fluctuations, a very long process, that that might have turned you off?*
Patient 4: *I would have still gone ahead with it, but I would have been extremely upset about it and it would not have helped me at all. In fact, I don’t think you should tell people. It may not be as hard for everybody else anyway.*
**Knowledge transfer**
Patient 1: *It is an onerous thing to pursue, it really is. You really would not want to be doing it lightly and you would not want to be doing it if you had other kind of reasonable alternatives. But that said, 15 years of depression is pretty jolly onerous too. It’s a little bit difficult to disentangle.*
Patient 5: *[You probably need to know] that it is a long process. You probably don’t realise probably just how long—but to be well informed. I think to be well informed, which if you’re going through the DBS program and you go to that panel, they question you a lot so you have to be informed…I think if people are like me, I find it hard retaining things and that sort of thing but I really had to work at being informed and to understand everything.*
Patient 6: *I think if I had to impart one piece of knowledge or advice to somebody starting off contemplating DBS, is to be prepared for a long, drawn-out process. It’s just not going to happen overnight, and these things take on a character all of its own. There’s nothing you can do as an individual to hasten the process. You’ve got to wait…everybody’s working very, very hard to get you there, but it just doesn’t happen overnight.*
Caregiver 6: *If they were in the situation as me, I’d say actually, go for it…I’ve got no regrets, and we’d exhausted everything we could think of…I mean, I think it’s something you’ve got to think about seriously, and ask all the questions, and there’s plenty of opportunity to do that. Don’t expect electric light overnight…it’s not just like changing a course of something for something else. It is—you could almost say a lifestyle change, because…once it’s there, it’s there, to the best of my knowledge…It’s going to be part of you, and you’ve got to live with it, and you’ve got to live with that recharging…But do sit down and talk about it and think it through. Because it’s not like putting on a new pair of shoes, you are having brain surgery.*
**No regrets**
Interviewer: *Knowing what you know now and what your outcomes have been, would you undergo the DBS surgery again if you had decision to make again?*
Patient 1: *Yes, I think I would. I suppose precisely because when there are no other alternatives the only thing you can do is roll the dice.*
Patient 2: *I was sort of hoping that it’s going to fix it straight away, and then when it didn’t…it was like, ‘oh maybe I shouldn’t have done it’, because it was quite stressing, as it all got toward it…to go through all that—the torture of the surgery—but then as it improved, I’m sort of thinking, I’d recommend it…Even though stressful in the process and very long…Yeah. I mean, it was all worth it.*
Patient 5: *Would I do it [again]? I think that’s a hard question to answer because it’s still in the process. I might say to you today I don’t know that I would but then things might change in a month or two months or three months. I said I wouldn’t have the surgery again, but if there was the possibility of something I probably would.*

### Anticipated vs. Actual Outcomes

In anticipation of DBS, participants shared beliefs about potential responses to stimulation. Most recognized that responses could vary considerably person-to-person and rather than being a one-off procedure, much testing and talking was needed to optimize settings. Some patients felt they would perceive no difference in stimulation settings unless one was exerting a beneficial effect. Others flagged potential “strange problems” e.g., hypomania, impatience or becoming “too over-reactive.” The prospect of the inactive (control) setting was particularly worrying for some, but was understood as a research requirement. The anticipated time it would take for patients to detect a beneficial setting varied from fairly immediate (as heard in “miracle” stories) (Racine et al., 2007), to a couple of weeks or months. One patient felt confident they would know if the treatment was working, having experienced distinct, albeit brief, periods of wellness in response to other treatments. In contrast, one caregiver was concerned their loved-one may not recognize improvements they were experiencing, as had occurred with another experimental treatment.

Participants distinguished between what they were expecting from the procedure and what they hoped the outcome would be. As an experimental trial, some held no expectations at all or considered their probability of remission in light of outcomes from other trials. With extensive histories of non-response to standard depression treatments, some were inclined to consider “no benefit” the most likely outcome and entertained few hopes to avoid later disappointment. In contrast, one patient described their expectations in positive, absolute terms (“I expect to recover”), explaining optimism was necessary to give them determination to proceed. This affirming mindset appeared balanced by a realistic understanding of the trial’s experimental nature. One patient and caregiver had held initially high expectations after seeing a positive case study in the media; however, these were tempered after discussions with the clinical trial team and receiving further information. Many emphasized how meaningful even a small or partial improvement would be for their quality of life.

In addition to DBS, participants were aware that engaging in additional therapies and practices would likely be required to aid recovery. Continuation of current approaches e.g., medication, psychotherapy, routine exercise etc. was still considered necessary to maximize rehabilitation and recovery. Some felt they would need to re-focus on lifestyle factors, including sleep hygiene, diet, exercise, and social connection. New input from counseling or social work services was recognized by participants for supporting psychosocial rehabilitation, e.g., regaining life-skills, re-establishing spousal relationships.

At the follow-up interview, majority of participants indicated their life after DBS had felt like “a roller-coaster ride.” This was regardless of their subjectively perceived outcome; meaningful improvement (*n* = 2), little (transient/subtle) to no benefit (*n* = 3), or increased depression (*n* = 1). Participants described a variety of responses they thought were due to stimulation settings. Positive changes included: a lift in mood (subtle to substantial), expressions of joy (tears/laughter), improved sleep, increased energy, more interest in people and surroundings, more talkative and engaged, and increased motivation to do things (shopping, create things, see people). In two cases, positive stimulation effects were sustained, while others only experienced transient benefits (2–3 days). Undesirable responses that participants attributed to stimulation settings were: increased irritability, anxiety, urges to self-harm, cognitive effects (confusion, poor memory and problem-solving), manic episodes, disturbed sleep, acne, and further decreases in mood, energy, motivation, and confidence. A general sense of unease (“off kilter,” “really crook”) was also common. These experiences were mostly transient and remitted with stimulation parameter adjustments. Turning the device on and off (in order to conduct medical procedures or patient self-experimentation with the device) was associated with: panic attacks, dissociative experiences, sensations of a childhood memory, and manic episodes. Other experiences, some related and others unrelated to DBS, also contributed to the roller-coaster experience, including surgery-related anxiety and trauma, adjusting to new emotions (joy, anger, pride), suicide attempts (*n* = 2), managing social reintegration, relationship difficulties (with caregiver and non-caregivers), medical issues, and family bereavement.

When reflecting how outcomes compared with initial expectations, most felt their situation was still a “work in progress.” Some who had only experienced glimpses of a positive effect remained hopeful that effective device settings could be identified. However, the question of how long to persist was raised. One patient who had no existing expectations was “neither disappointed nor surprised” with their apparent lack of benefit. Those who had experienced benefit were in the infant stages of wellness, navigating various new experiences. In one case psychological support was important for guiding the patient’s adjustment. Some participants offered percentage indicators of recovery (varying from 40 to 70% improvement). While these exceeded prior comments that “any improvement” would do, a desire for more to be gained post-trial was present. One patient who became interested in DBS after viewing a positive media story expressed disappointment that they had not experienced such an immediate or dramatic effect.

### Trial Decision-Making and Knowledge

In anticipation of surgery, candidates discussed their decision to pursue DBS. Most were introduced to DBS by their treating psychiatrist, where it was raised as an experimental approach being trialed for treatment-resistant individuals. Others encountered it while researching alternative treatments online. All had treating clinicians willing to support their application into the clinical trial. All patients had conducted some personal research into DBS, including reviewing the peer-reviewed literature (those with higher education, academic experience) to searching Google and YouTube (case studies, footage of surgery). This research was mostly driven by patients themselves, but occasionally by caregivers with patients then viewing their findings. Some caregivers conducted limited research, as they trusted the patient’s judgment and the experts supporting them. While independent research and discussions with medical professionals were influential, the lack of treatment alternatives was the driving force behind most patients’ decision. All felt confident they had exhausted all alternative treatment options and while DBS was considered “pretty hard core,” as the only option left, patients were willing to pursue something “extreme.” Being approved for DBS provided hope some caregivers felt was keeping their loved one from suicide.

Participants recalled many surgical risks associated with DBS (e.g., stroke, brain bleed, infection, seizure, general anesthetic complications, death). While concerning, most were comforted knowing it was a routine surgery for movements disorders (i.e., Parkinson’s disease, essential tremor) with low chance of adverse events, and had trust in the surgeon’s skill. The surgical risks were often compared with those associated with undergoing ECT, with hope DBS would reduce future need for ECT. Awareness of stimulation-related adverse events appeared less well known, although mania/hypomania, sleep disturbances, and adverse mood effects (anxiety, agitation) were raised by some. One caregiver considered these a part of the research process for finding the best settings, while another expressed concern their loved one would not report adverse effects and attempt to endure them.

Participants generally felt well-informed of the risks and benefits, having held multiple discussions with the clinical trial team, the surgical team, and their treating clinician over many months and years. One patient noted they “had to be” well-informed in preparation to sit before the Victorian Mental Health Tribunal. Some patients felt having an opportunity to talk to others with a DBS implant (not necessarily for depression), would be beneficial to understand more about the physical aspect of having the device.

After DBS, participants reflected on how informed and prepared they had been prior to surgery. Some patients were surprised by the physical discomfort resulting from the implanted device e.g., tenderness behind ear where wire runs, pain in chest where implantable pulse generator (IPG) rests when sleeping on side and while driving due to seatbelt rubbing against IPG. While acknowledging the difficulty in pre-empting such outcomes, one patient felt they would have preferred the IPG on the opposite side had they been given a choice. And while prepared for surgery from a procedural perspective, one patient felt unprepared for how personally confronting the experience was, and highlighted the importance of comfort and reassurance from medical staff during the procedure. Participants generally felt well-informed regarding risks and procedures, but were surprised by how long the whole process was (e.g., getting Tribunal approval, scheduling surgery, conducting clinical trial tests, and regularly adjusting parameters). The number of research center visits required had also been a surprise to some, with both caregivers and patients acknowledging they may have not fully absorbed this information prior.

Participants were asked what advice they would give others considering DBS for depression based on their experience. The most common perspective was that it must be a last resort and all less invasive options need to be tried first. Being well-informed and prepared was considered essential, while remembering it is a research trial and a positive outcome, like you might read or hear about, is unlikely. Being prepared for a “long, drawn-out process” was also emphasized. Many felt that if someone (patient or caregiver) were “in the same boat” as they had been and were considering DBS, they would recommend it, even if their outcome had not been overly positive.

Participants were also asked to consider if they had their time again, would they still decide to undergo DBS (or support their loved-one to)? Most participants felt they would, regardless of their experience and present outcome. The lack of alternatives was again sighted as a worthy reason to attempt it, and those who had experienced no benefit felt their situation could change, so it was yet to be determined whether it was worthwhile.

## Discussion

Through prospective semi-structured interviews with key stakeholders, this study sought to gain in-depth knowledge and insight into the lived experience of DBS for TRD. Through this process, key ethical issues were explored including: informed consent, decision-making capacity, intervention expectations, and subjective outcomes. The relevant findings and implications of each are discussed below.

### Informed Consent and Decision-Making Capacity

In most instances, participants demonstrated reasonable knowledge and understanding of DBS for TRD, including awareness of potential risks and benefits. Patients reported feeling both well-informed and prepared, having participated in extensive consenting discussions. All had been evaluated by the Victorian Mental Health Tribunal in order to receive permission to undergo DBS. This process involves a hearing held between three tribunal members, the DBS candidate, a support person (if needed), and the clinical/research team. The panel assesses the candidate’s suitability for the procedure and capacity to consent to it. The majority of the candidates indicated that they found this experience anxiety-provoking and onerous. It could be argued that this requirement stigmatizes those with mental illness compared with other neurological indications for DBS where neurocognitive impairment is common (i.e., Parkinson’s disease) (Thomson et al., 2019a). While the efficacy of DBS for depression remains under investigation, however, this safeguarding procedure should ensure that only people with acceptable levels of understanding and preparedness who are well supported by an appropriately knowledgeable and experienced clinical team proceed to surgery.

Despite demonstrating comprehension and retention of information required to make an informed decision, some post-DBS comments indicated that this had little bearing on the candidates’ decision and acknowledged the impact depression had on their engagement with this information (e.g., impaired memory, concentration difficulties, challenges making independent decisions, hopelessness/nihilism). Hopelessness, suicidal ideation and reduced concern for preservation of ones’ life (“I want to die anyway” Patient 4) affected appraisals of surgical risks and desperation to be relieved from persistent depression in the absence of alternatives meant participants were willing to take a chance. Others have noted the difficulties involved in establishing meaningful informed consent for DBS in the midst of extreme hopelessness, desperation, and a lack of alternatives (Bell et al., 2010; Klein et al., 2016). Given the severity of mental illness, poor quality of life, lack of treatment prospects, and risk of dying by suicide possessed by people with TRD, it is reasonable and expected that their appraisals of risk and decision-making will be influenced by hopelessness and nihilism. These inherent features of TRD should be recognized, acknowledged and balanced alongside persistent efforts to conduct thorough and comprehensive consent. Further patient-led research is needed to understand how best to provide information about DBS and maximize participant comprehension and appreciation, particularly of long-term risks and outcomes.

Participants were aware of the short-term risks (i.e., risks posed by undergoing surgery). DBS surgical risks are well-documented, and given their life-threatening potential, it is unsurprising that these were in the forefront of participants’ minds during pre-DBS interviews. Less certainty was expressed regarding stimulation and device-related risks. This aligns with findings in Parkinson’s disease, where patient and caregiver awareness of surgical risks was superior to stimulation-related risks such as transient personality and behavioral changes (Thomson et al., 2020). Patients in the current study experienced various stimulation-related effects that remitted following adjustment (e.g., anxiety, irritability, disturbed sleep, mania, self-harm urges). While transient, some of these were unsettling and distressing for participants, contributing to their “rollercoaster” experience. Uncharacteristic and problematic stimulation-dependent behaviors (e.g., impulsive and reckless decision-making while manic) also have the potential to impact the individual and their relationships in the long-term (Agid et al., 2006; Mosley et al., 2019; Thomson et al., 2019b).

Participants demonstrated some awareness, but ultimately an underappreciation of, long-term risks and consequences of DBS. Such consequences included the time-burden associated with regularly recharging the DBS device (discussed at length in Thomson et al., unpublished) and the travel/time-burden associated with frequent visits to the research center (to complete clinical tests, stimulator adjustments and monitor effects). This was particularly the case for those living interstate and caregivers with work commitments. Full pre-surgical appreciation of these long-term implications appeared inhibited by the urgency to pursue a novel treatment. When people express their situation “can’t get any worse” or they “have nothing to lose” (Caregiver 6), emphasizing how participation in a clinical trial and receiving DBS could alter and complicate daily life is particularly important (de Haan et al., 2015; Thomson and Carter, 2020). As one caregiver described it—DBS is “a lifestyle change.” While patient and caregiver journeys with DBS were rarely straightforward, all indicated they would make the same decision if they had their time again. For the reasons that: (1) they had derived some benefit from DBS, (2) they felt hopeful they may yet still, or (3) in order to have the question “will it work?” answered.

### Intervention Expectations

High expectations have been highlighted as another aspect of informed consent that require careful management given the potential for disappointment and negative postoperative outcomes (Bell et al., 2009, 2010). In other populations (Parkinson’s disease, epilepsy), unrealistic expectations have been associated with poorer psychosocial adjustment and subjective, psychometric and functional outcomes (Wilson et al., 2001; Haahr et al., 2010; Maier et al., 2013; Hasegawa et al., 2014; Baertschi et al., 2019). Media stories are often implicated in the development of unrealistic expectations. Media portrayals of DBS are overwhelmingly positive, depicting best-case scenarios with limited reporting of associated risks (Racine et al., 2007; Gilbert and Ovadia, 2011). Media coverage of DBS for depression generally presents the “miracle cure” narrative (Dobbs, 2006; Talan, 2008; CNN, 2014; PBS, 2016). The other extreme, the “horror story,” is depicted to a lesser extent (Egan, 2015). Such coverage can result in blind optimism or unfounded fears of DBS, both of which have the potential to damage the scientific development of the emerging intervention (Johansson et al., 2011). In one couple’s case, their decision to pursue DBS had been influenced by a positive media story. Despite having gone through a comprehensive consent process about the procedure’s experimental nature, they still experienced disappointment with an inadequate outcome. This demonstrates the potency of hope elicited by such narratives, and reflects what Baertschi et al. (2019) refer to as the *emotional* facet of expectations.

Some participants expressed strong hopes for DBS that were held in balance with knowledge and understanding of the intervention’s experimental nature and uncertain outcome. Based on interviews conducted with a sample of DBS recipients with Parkinson’s disease, Baertschi et al. (2019) identified participant expectations consisting of two distinct components. Some patients could intellectually acknowledge the science and research-based information reinforced by medical professionals (*cognitive* facet), while still holding hopes the treatment would lead to something extraordinarily positive (*emotional* facet). The authors report that these “secret hopes” were not revealed to medical staff pre-operatively. In the current study, hope appeared to be a powerful motivator to proceed with the experimental procedure and intensive clinical trial, regardless of how small or unspoken it was. The degree of hope present in participants’ pre-DBS mindsets varied and appeared to serve a protective function. Some participants maintained an optimistic and hopeful mindset in order to have the courage and motivation to follow through with the intervention. While others maintained a rational mindset with minimal acknowledgment of their hopes, for fear of later disappointment (as had been experienced numerous times before). A hopeful mindset is not necessarily problematic (Sotsky et al., 2006), unless of course it reflects a fundamental misunderstanding of the research purpose or prospect of benefit (Horng and Grady, 2003). Indeed, hopelessness (a common symptom of depression itself), has the potential to obscure perceptions of benefit and intervention outcomes (Brent et al., 1998).

### Subjective Outcomes

Participants reflected extensively on their experience of living with DBS, including the perceived benefits of the procedure (or lack thereof). The significance of small changes was evident. For example, a patient was relieved to spend minutes rather than hours crying upon waking each morning, and a caregiver was thankful to be able to engage in small conversations with their partner rather than sitting in silence. Such changes were considered meaningful and while many pre-DBS comments suggested that *any* improvement would do, a desire to gain more from DBS was common. In other DBS samples (movement and psychiatric disorders), there is evidence that patients and caregivers shift their expectations for DBS based on their postoperative experiences. This includes patients wanting to achieve goals that were not discussed preoperatively or that medical professionals indicated were unattainable [e.g., “(The patients) shift the goalposts a little” DBS Nurse] (Thomson, 2020). Patients’ desired level of control over their DBS stimulator can also alter as they adjust to living with the device (Merner et al., 2021). Families can also develop an increased expectation that adjustment of the DBS settings will resolve any issue they observe in the patient (Klein et al., 2016), a perception that can be frustrating and invalidating for the patient themselves.

Another common reflection from participants was the length and uncertainty of the process of establishing whether DBS had “worked.” DBS for any treatment indication requires extensive testing and trialing of stimulation parameters, a process that can expose patients and families to a variety of desirable and undesirable changes. This “rollercoaster” experience of encouraging and disappointing responses was a surprise to most participants, as was the length of the optimization process. In comparison with movement disorders, the process of optimizing settings in depression is complicated by the lack of consistent acute behavioral and clinical effects (Ramasubbu et al., 2013), with a lag of 2 weeks between adjustment and detectable effects common (Holtzheimer et al., 2012). Regardless of clinical trial protocols, optimization is a complex, time-consuming, and at times imprecise process that can take 12-months or more to complete (Dougherty et al., 2015; Bergfeld et al., 2016; Ramasubbu et al., 2018; van Westen et al., 2021b). While participants were prepared for this, the reality of the process was challenging. Adjusting to the observed changes is rarely straightforward either. In OCD, DBS patients often take time to recognize changes, before gradually making sense of them and integrating them successfully into their lives (de Haan et al., 2015; van Westen et al., 2021a). As one patient with OCD remarked: “DBS is no ON/OFF switch” (p. 12) (van Westen et al., 2021b). It is therefore unsurprising that at 9-months post-DBS many of the current sample considered DBS “still a work in progress” (Caregiver 3).

### Implications and Recommendations

The current findings hold a number of implications for informing and consenting participants to clinical DBS research. There have been calls from both neuroethics and scientific communities for the long-term risks and consequences of participation in psychiatric DBS trials to be robustly outlined for potential participants (Hendriks et al., 2019; Goering et al., 2021; Vedam-Mai et al., 2021). This would include thoroughly addressing the burden associated with participation (regular travel to research center, clinical tests), information on the day-to-day impact of DBS (e.g., recharging, stimulation side-effects, psychosocial adjustment), and providing clear guidance on post-trial continuity of care (given DBS can be a life-long intervention that is dependent on specialist care and requires maintenance) (Thomson and Carter, 2020). There are limitations to contractual “disclose and sign” informed consent processes, notably that information presented preoperatively is later forgotten or goes unappreciated. There have been recommendations for more experiential and interactive forms of informed consent in DBS (Bell et al., 2010; Liddle et al., 2019), that draw upon the knowledge of lived experience experts. Such a process could involve corrective feedback and use of narrative accounts from DBS recipients and their families (e.g., videos, written accounts). Those with lived experience can answer questions and provide perspectives that clinical research teams cannot. Information delivered in narrative form is also often well-retained (Mazor et al., 2007; Thomson et al., 2020). Further research is required to establish what forms of narrative evidence are most effective; however, a range of different outcomes and experiences must be represented to ensure personal stories do not set an expectation for a single, best-case scenario (as occurs with media stories). The current findings also demonstrate that participants’ desires and expectations for DBS adjust based on their personal experience with the device. As such, informed consent for this long-term and adaptable neurotechnology requires an iterative and ongoing process. This recommendation has previously been made for DBS in Parkinson’s disease (Kubu et al., 2018; Liddle et al., 2019; Mosley et al., 2019), and is potentially more relevant in clinical trials of DBS for psychiatric conditions where the risks and benefits are less established.

A further recommendation is for an expansion of DBS clinical trial protocols to include more in-depth, qualitative studies of this kind. Data collected in clinical trials provides important indicators of intervention efficacy and safety; whereas qualitative data provides insight into the experiential effects of DBS and can elucidate unexpected or paradoxical outcomes (e.g., difficulties with psychosocial adjustment). Thus, adopting both approaches will give the most complete picture of the impacts of DBS. The variety of experiential information derived from qualitative studies can be used to better inform prospective patients and caregivers on what to expect. Qualitative research strives for transferability rather than generalizability, and an overview of patient experiences can assist in preparing patients and families for DBS.

A final recommendation is for increased inclusion of caregivers in the research process. This includes informed consent procedures in order for caregivers to have a full understanding of what their loved one is agreeing to and what impact it is likely to have on both of their lives. Research teams often seek informal feedback from caregivers about what they are observing in the patient, whether it be subtle improvements (e.g., increase in energy, activity) or excessive adverse effects (e.g., mania). There is, however, much potential in examining how caregivers themselves are affected by their loved one’s participation in a DBS clinical trial (e.g., quality of life, mood and anxiety).

### Limitations

The patient sample was small (*n* = 6) and reflects the small numbers undergoing the procedure for depression in Australia. This sample represents the entirety of those who have received DBS for TRD in Australia since December 2016. For the purpose of deriving in-depth, qualitative information, samples of this size can be sufficient (Crouch and McKenzie, 2006; Guest et al., 2006) and data saturation (commonly used to determine adequate sample size) was reached. This rich and in-depth data has high transferability; however, the specific context in which this data was derived should be held in mind (e.g., target lead location, patient characteristics, geographical location, clinical trial protocol, available psychosocial supports etc.) given the potential for such factors to influence experiences and outcomes.

## Conclusion

This is the first prospective qualitative study to be conducted with individuals undergoing DBS for TRD, with the added perspective of caregivers. The prospective design ensured participants’ knowledge, expectations and beliefs accurately reflected their pre-DBS circumstances and allowed for contrast with actual outcomes and experiences. Caregivers played an important role throughout the DBS process and were impacted by their loved one’s participation in various ways. The progress and development of psychiatric DBS clinical research depends on knowledge acquired through both large-scale, robust clinical trials as well as small, in-depth qualitative studies such as this one.

## Data Availability Statement

The datasets presented in this article are not readily available because of the personal nature of the information included within the interview transcripts and in order to maintain participant confidentiality. All relevant data are presented within the manuscript. Requests to access the datasets should be directed to corresponding author.

## Ethics Statement

The studies involving human participants were reviewed and approved by the Monash University Human Research Ethics Committee. The patients/participants provided their written informed consent to participate in this study.

## Author Contributions

CT, RS, ER, and AC designed the study and research protocol. CT recruited study participants, collected the interview data, and analyzed the interview data. AC and RS assisted with data analysis interpretation. CT wrote the manuscript. All authors provided critical feedback and contributed to the article and approved the submitted version.

## Conflict of Interest

PF has received equipment for research from MagVenture A/S, Nexstim, Neuronetics and Brainsway Ltd., and funding for research from Neuronetics. He is a founder of TMS Clinics Australia. The remaining authors declare that the research was conducted in the absence of any commercial or financial relationships that could be construed as a potential conflict of interest.

## Publisher’s Note

All claims expressed in this article are solely those of the authors and do not necessarily represent those of their affiliated organizations, or those of the publisher, the editors and the reviewers. Any product that may be evaluated in this article, or claim that may be made by its manufacturer, is not guaranteed or endorsed by the publisher.

## Footnotes

1 Inclusion/exclusion criteria, extensive demographic data, surgical information (including lead placement), and full psychometric outcomes from the clinical trial will be reported in a subsequent publication (in preparation). Any correspondence regarding this efficacy study should be directed to paul.fitzgerald@monash.edu
